# From Nanorods to Nanowires of CdS Synthesized by a Solvothermal Method: Influence of the Morphology on the Photoactivity for Hydrogen Evolution from Water

**DOI:** 10.3390/molecules21040401

**Published:** 2016-03-24

**Authors:** Fernando Vaquero, José Luis G. Fierro, Rufino M. Navarro Yerga

**Affiliations:** Instituto de Catálisis y Petroleoquímica, CSIC, Marie Curie 2, Madrid 28049, Spain; f.vaquero@csic.es (F.V.); jlgfierro@icp.csic.es (J.L.G.F.)

**Keywords:** CdS, photocatalyst, hydrogen production, solvothermal synthesis, nanorods, nanowires

## Abstract

The effect of temperature and water/thiourea ratio on the growth, crystallinity and morphological characteristics of CdS nanostructures synthetized by a solvothermal method using ethylenediamine as solvent were studied. The temperature and water/thiourea ratio used in the synthesis determine the surface area, shape, length and degree of crystallinity of the CdS nanostructures obtained. Nanowires of high crystallinity and length were obtained when the solvothermal synthesis was performed at 190 °C, while nanorods with lower length and crystallinity were obtained as the solvothermal temperature decreased to 120 °C. The change in the water/thiourea ratio affects the crystallinity and length of the CdS nanostructures to a lesser extent than temperature. Nevertheless an increase in the water/thiourea ratio used during the solvothermal synthesis resulted in CdS nanorods with higher crystallinity, lower aspect ratio and lower specific surface area. Textural, structural and surface properties of the prepared CdS nanostructures were determined and related to the activity results in the production of hydrogen from aqueous solutions containing SO_3_^2−^ + S^2−^ under visible light.

## 1. Introduction

The conversion of solar energy into hydrogen via the water splitting process assisted by photosemiconductors is one of the most interesting ways to achieve a clean and renewable energy system [1,2]. Cadmium sulfide (CdS) nanostructures have been widely applied for solar driven H_2_ production due to its suitable band gap (2.4 eV) and band gap energy [3,4,5,6,7,8,9,10,11]. The photoactivity of CdS nanostructures depends on their crystalline phase, structure, morphology, defects and size. In the nano regime, the photoactivity of CdS was improved in samples with wurtzite structure, good crystallinity and short bulk-to-surface diffusion distance for photogenerated electrons and holes [4]. Taking into account the effects of these factors on the photoactivity of CdS nanostructures, much effort has been made to control the synthesis of CdS at the nanometric scale [4,9,12]. In particular, zero-dimensional (0D) quantum dots and one-dimensional (1D) CdS nanostructures such as nanorods, nanowires and nanoribbons have received considerable attention in the last years due to their potential to enhance photoactivity with respect to bulk CdS [13,14,15,16]. Quantum dots and 1D CdS nanostructures show a strong quantum confinement effect (SQE) when the nanostructures have a diameter lower than the exciton Bohr radius of CdS (2.5 nm) [17]. These nanostructures have several interesting advantages such as high surface-to-volume ratios, quantum confinement effect, as well as slow electron-hole recombination. Among the preparation methods explored to prepare crystalline CdS nanostructures with controlled morphology, the solvothermal method is a relatively simple methodology which allows to obtain crystalline nanostructures of CdS at moderate temperature and pressure [18,19,20,21]. Highly pure 1D CdS nanostructures with a high degree of crystallinity can be produced by the solvothermal route using ethylenediamine or other amines as solvents [22]. To date, although CdS nanostructures with different structure, size and morphology have been synthesized by the solvothermal route, the influence of CdS morphology and size on photoactivity for hydrogen production have received only limited attention. For example, in the study of Yu *et al.* [23] the better photoactivity of CdS nanowire structures than that obtained on nanorods or CdS nanoparticles was shown. On the other hand, Lang *et al.* [24] reported high photocatalytic hydrogen production on multi-armed CdS nanorods attributed to the positive synergistic effects of the hexagonal phase and the morphology of CdS, while Jang *et al.* [25] reported that CdS nanowires with higher crystallinity showed a higher rate of photocatalytic hydrogen production. In spite of these studies, there is no clear correlation between the characteristics of solvothermal CdS nanostructures and their photoactivity. Taking into account that the size and shape of CdS nanostructures synthesized by solvothermal methods depend on the solvent, the metal and sulfur source, the temperature and the time [18,19,26,27], in the present work we studied the effect of temperature and water/thiourea ratio on the growth, crystallinity and morphological characteristics of the resulting CdS nanostructures. The changes in the solvothermal variables lead to CdS nanorods and nanowires with different size that allowed elucidating the consequences of the structural, morphological and surface characteristics of these nanostructures on photophysical properties as well as in the photoactivity for hydrogen production under visible light form aqueous solutions containing Na_2_SO_3_ and Na_2_S as sacrificial electron donor agents.

## 2. Results

### 2.1. Physicochemical Characterization

#### 2.1.1. TXRF and Textural Analyses

According to the temperature and water/thiourea ratio used in the solvothermal synthesis samples were labeled as CdS-*x* (*x* = 120, 150 and 190 °C and 1.3, 2.0 and 3.0 water/thiourea molar ratio). Surface chemical analysis by TXRF indicates the existence of only Cd and S in all prepared CdS samples (Table 1). All samples, except CdS-150 and CdS-3.0, have similar surface composition and coordination of Cd with S. The Cd/S atomic surface ratio was close to 0.85 in all samples, except in the samples CdS-150 and CdS-3.0 for which the ratio increases to a value close to 0.90.

**Figure 1:**
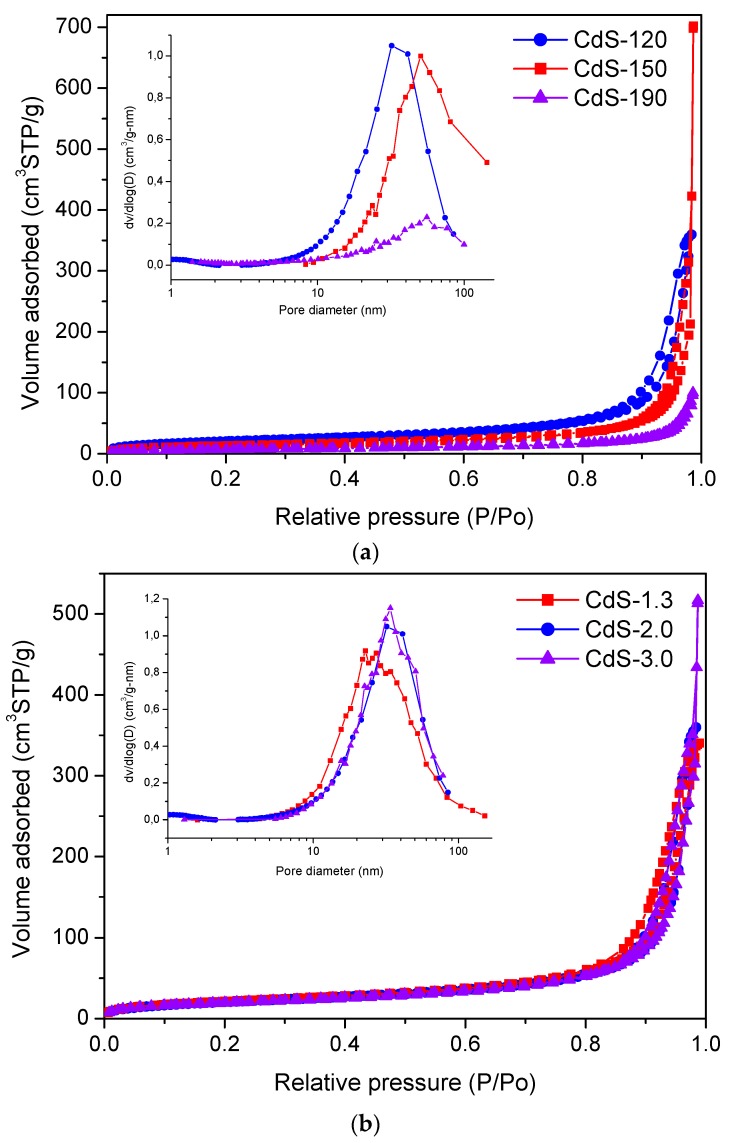
N_2_ adsorption-desorption isotherms and the corresponding pore size distribution curves (inset) of the CdS samples: (**a**) influence of solvothermal temperature (CdS-120, 150 and 190); (**b**) influence of H_2_O/thiourea ratio (CdS-1.3, 2.0 and 3.0).

The nitrogen adsorption-desorption isotherms and the corresponding pore-size distributions of as-prepared CdS samples are presented in Figure 1. All the samples displayed type IV N_2_-isotherms (IUPAC classification) characteristic of mesoporous materials with low contribution of micropores [28,29]. All isotherms displayed hysteresis loops of type H3, associated with the presence of narrow slit-shaped pores due to the aggregation of CdS nanostructures. The pore-size distribution curves (inset in Figure 1) of CdS samples are very broad, further confirming the presence of mesopores [30].

Textural data of CdS samples (Table 1) show that their specific surface area varies with the solvothermal temperature and water/thiourea ratio used in the synthesis. It is observed that the specific surface area of the CdS samples drops as the solvothermal temperature and water/thiourea ratio increase. This decrease is assigned to the collapse of the mesopore structure of the samples associated with changes in the size and morphology of the CdS particles induced by the temperature and water/thiourea ratio used in the solvothermal synthesis.

#### 2.1.2. Powder X-ray Diffraction (XRD) Analysis

**Figure 2:**
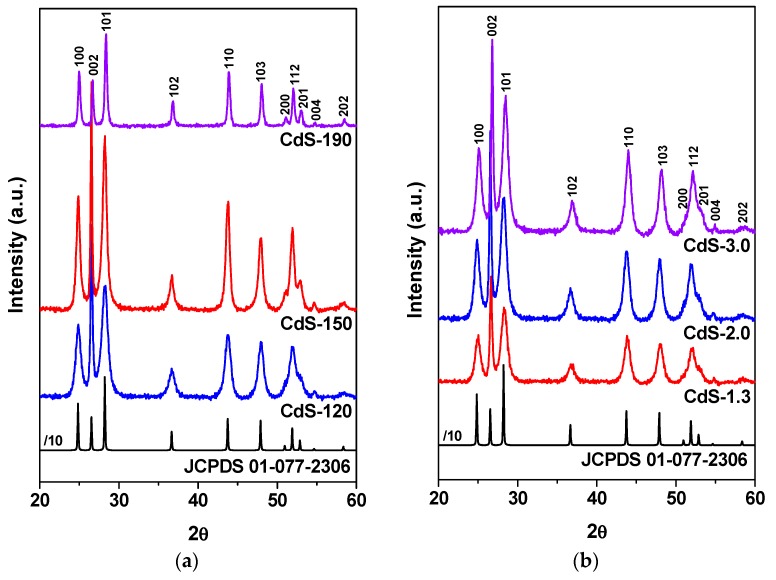
Powder XRD patterns of CdS samples: (**a**) influence of solvothermal temperature (CdS-120, 150 and 190); (**b**) influence of H_2_O/thiourea ratio (CdS-1.3, 2.0 and 3.0).

Figure 2 displays the XRD diffraction patterns of CdS samples. All patterns displayed only reflections corresponding to the formation of CdS with hexagonal crystal structure (JCPDS 01-077-2306) with P63mc space group and lattice constants of a(Å) = 4.136 and c(Å) = 6.713. No peaks corresponding to impurities were detected. The relative intensity of diffraction peaks changes with the solvothermal temperature evidencing differences in the morphology of CdS nanocrystals. In the case of CdS samples prepared at lower temperature (120 and 150 °C), the relative intensity of the peak corresponding to (002) planes was more intense than expected for the standard hexagonal pattern. This suggests that there is a relatively high crystalline order along the [001] direction (along the *c*-axis) indicative of the formation of 1D nanostructures. However, in the sample prepared at 190 °C, the intensities of (100) and (101) peaks increased, while that of (002) peak decreased. This represents the lower preferential orientation growth in the [001] direction of nanostructures as the solvothermal temperature increases above 150 °C. Upon increasing the solvothermal temperature, the crystallinity of the samples continuously improved as it can be seen by looking at the stronger and narrower diffraction peaks. Quantitative estimation of CdS phase crystallite sizes by applying the Scherrer equation (Table 2) has been calculated from the broadening of the (002) reflection of CdS phase (at 2θ angle of 26.5°). These values indicate that the crystallite size of CdS continuously increases when the solvothermal temperature rises up to 190 °C.

Figure 2 also includes the XRD patterns of CdS samples prepared with different water/thiourea ratio. It was observed that the increase of water concentration during the solvothermal synthesis results in a higher degree of crystallinity of the CdS nanostructures. This result indicates that the excess of water facilitates the crystallization growth of CdS. In these samples, the relative intensity of the peak corresponding to (002) planes was also more intense than expected for the standard hexagonal pattern and indicative of a preferential growth along the *c*-axis with formation of 1D nanostructures. The intensity ratio of (100)/(002) and (101)/(002) peaks does not change (Table 2) with the water/thiourea ratio which means no changes in the orientation of crystalline growth of CdS. The crystallite size of these CdS nanostructures (Table 2), slightly increases with the increase in the water/thiourea ratio used in the synthesis.

#### 2.1.3. FE-SEM Analysis

**Figure 3:**
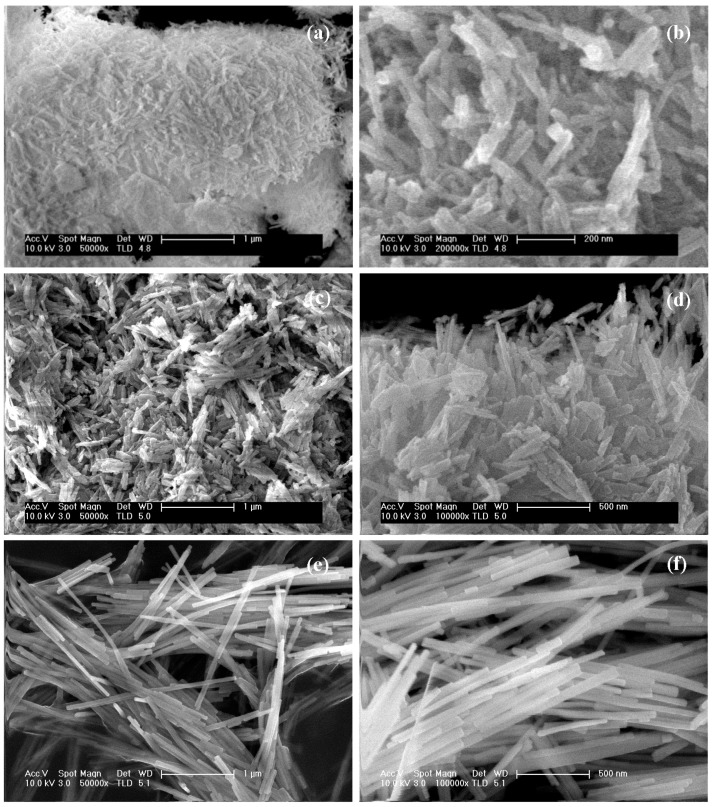
FE-SEM images of CdS samples prepared at different solvothermal temperature: (**a**,**b**) CdS-120; (**c**,**d**) CdS-150 and (**e**,**f**) CdS-190.

The FE-SEM images of the CdS samples synthesized at different solvothermal temperature, presented in Figure 3, showed changes in the morphology and size of the agglomerates of CdS particles (Table 3).

The sample prepared at 120 °C (Figure 3a,b) presents a mixture of CdS sheets with emerging irregular heterogeneously sized filaments on their surface. The sample prepared at 150 °C (Figure 3c,d) is made up of irregular clusters of heterogeneously sized filaments, from 0.7 to 12 µm, while the sample prepared at 190 °C (Figure 3e,f) shows better defined fibres of a few microns in length.

**Figure 4:**
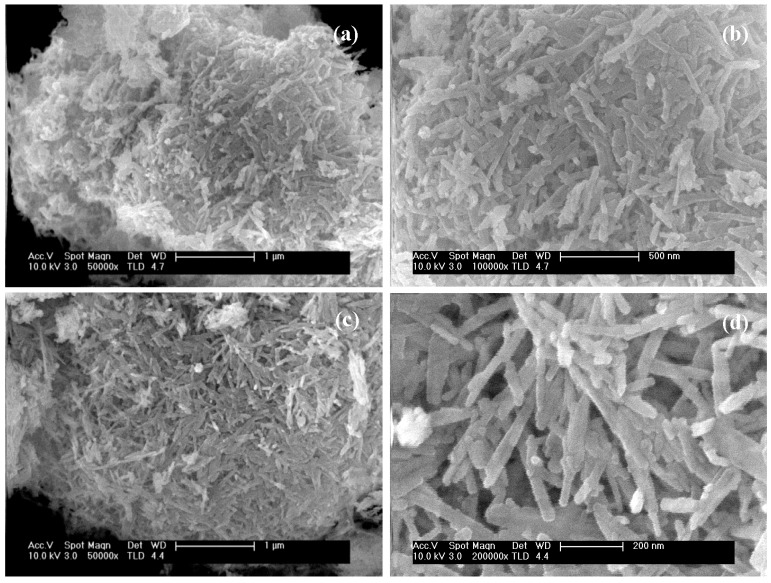
FE-SEM images of CdS samples prepared with different H_2_O/thiourea ratio: (**a**,**b**) CdS-1.3 and (**c**,**d**) CdS-3.0.

Figure 4 presents FE-SEM images of the CdS samples prepared with different water/thiourea ratio. The samples show incipient formation of nanorods on the surface of irregular nanosheet structures with a diameter of *ca.* 1.5–3 micrometers. As the concentration of water in the solvothermal synthesis increases, the samples present filamentous structures with more defined shape and crystallinity.

#### 2.1.4. TEM and HRTEM Analysis

**Figure 5:**
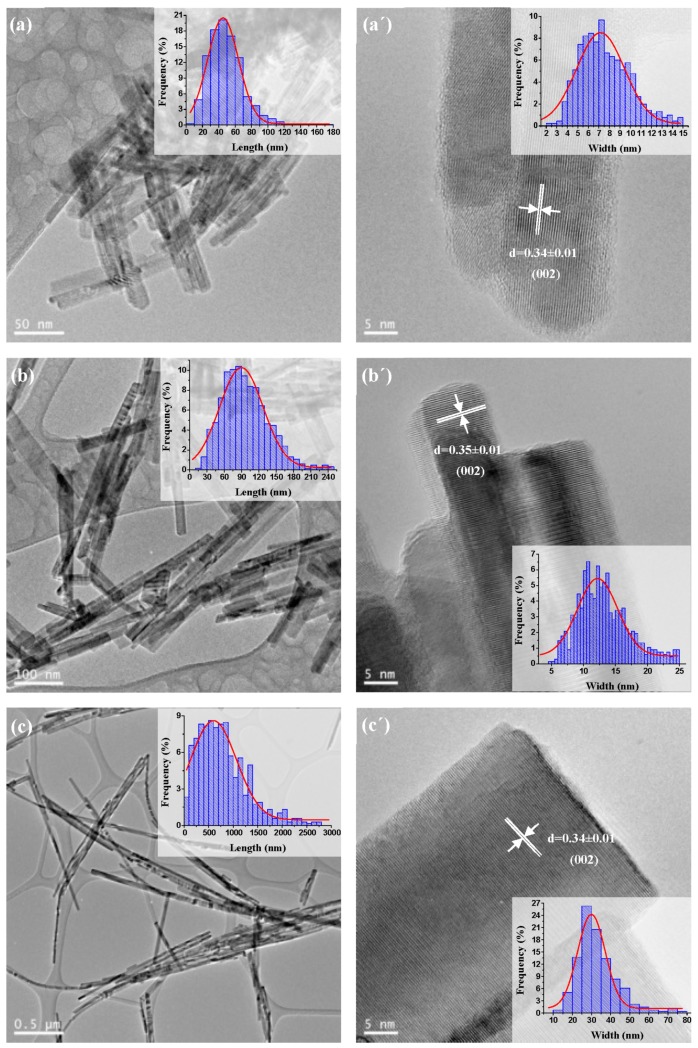
TEM and HRTEM images and size distribution curves (inset) of CdS samples prepared at different solvothermal temperature: (**a**,**a’**) CdS-120; (**b**,**b’**) CdS-150 and (**c**,**c’**) CdS-190.

The TEM images of CdS samples synthesized with different solvothermal temperature are shown in Figure 5 and the morphological characteristics of CdS nanostructures obtained from TEM are listed in Table 4. The TEM images confirm that the temperature used in solvothermal synthesis has a significant effect on the crystallinity, morphology, length and width of the nanostructures of CdS obtained. It can be observed that use of a higher temperature in the solvothermal synthesis affords 1D nanostructures with more homogeneous interplanar distance, longer length and more defined shape.

Sample CdS-120 (Figure 5a,a’) exhibits 1D nanostructures with an average size around 7 nm in width and 50 nm in length that leads to an average aspect ratio (length/width) close to 7, characteristic of nanorod structures. For the CdS-150 sample (Figure 5b,b’), the average length of the 1D nanostructures increased up to 90 nm and the width also increased up to 12 nm with an average aspect ratio of 7.5, close to that obtained for the sample CdS-120 and therefore characteristic of nanorod structures. Sample CdS-190 (Figure 5c,c’) showed more elongated 1D nanostructures with an average size around 700 nm in length and 30 nm in width, having an average aspect ratio of 23, higher than the value obtained in the samples CdS-120 and CdS-150 and corresponding to the formation of nanowire structures.

**Figure 6:**
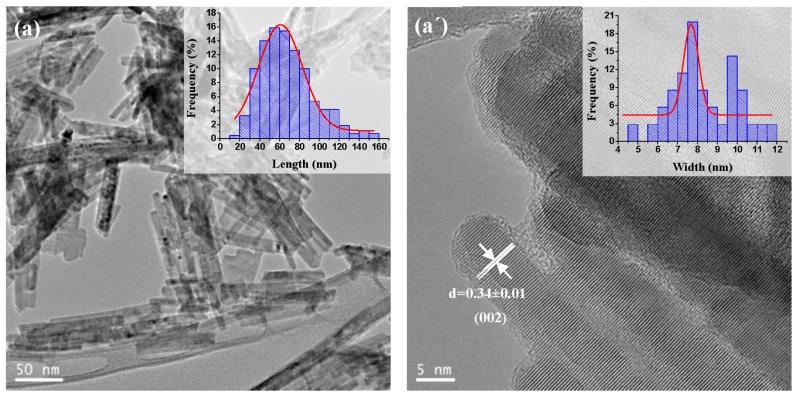

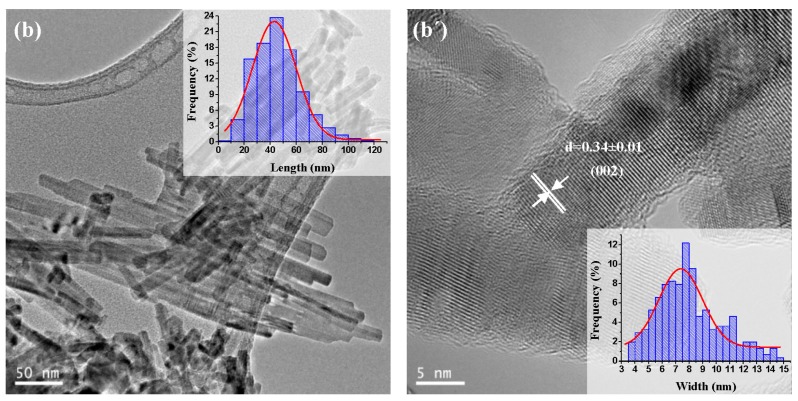
TEM and HRTEM images and size distribution curves (inset) of CdS samples prepared with different H_2_O/thiourea ratio: (**a**,**a’**) CdS-1.3 and (**b**,**b’**) CdS-3.0.

Figure 6 shows the TEM images of CdS samples prepared with different water/thiourea ratios. As shown in the TEM images, the water/thiourea ratio used in solvothermal synthesis has a moderate effect on the morphology and size of the resulting CdS nanostructures. It is observed that a higher water/thiourea ratio implies the formation of 1D nanostructures with more homogeneous interplanar distance and diameter and smaller length (Table 4). The sample prepared with lower water/thiourea ratio, CdS-1.3 (Figure 6a,a’), exhibits nanostructures with 60 nm average length and 7.5 nm in width that leads to an average aspect ratio equal to 8.0, characteristic of nanorod structures. The sample obtained with a higher water/thiourea ratio, sample CdS-3.0 (Figure 6b,b’) shows shorter nanostructures with 45 nm average length and 7.2 nm in width, having an average aspect ratio of 6.2 indicative of the presence of nanorods.

#### 2.1.5. UV-Vis Diffuse Reflectance Spectra Analysis

**Figure 7:**
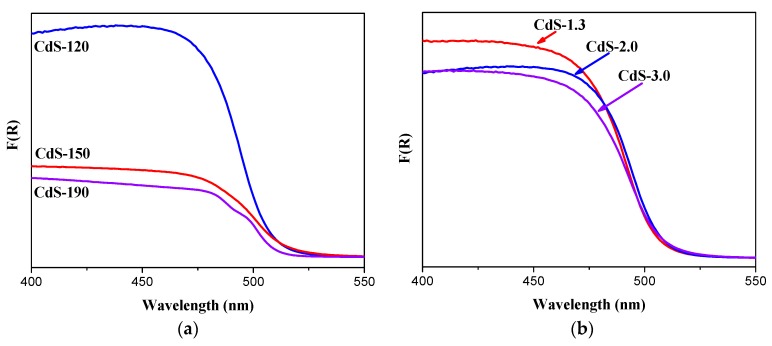
UV-Vis spectra of CdS samples: (**a**) influence of solvothermal temperature (CdS-120, 150 and 190); (**b**) influence of H_2_O/thiourea ratio (CdS-1.3, 2.0 and 3.0).

Diffuse reflectance spectra of all CdS samples are displayed in Figure 7. All samples show similar shapes in the absorption edges with a steep absorption edge that implies the only single CdS phase in all photocatalysts. The band gap energies (eV) of all CdS photocatalysts estimated from Tauc plots are in a close range of 2.45–2.50 eV as listed in Table 1.

All samples show a band gap slightly higher than that reported for bulk CdS (2.40 eV) indicative of a weak confinement effect associated to nanostructures of particle size slightly higher than the Bohr radius. The absorption spectra of the CdS samples prepared with different water/thiourea ratio (Figure 7) show similar absorption edge around 510 nm. Conversely, the absorbance intensity at a wavelength of less than 500 nm showed differences between the CdS samples synthesized at different solvothermal temperatures. The differences in the particle size of the CdS samples may contribute to the observed changes in the absorbance intensity because small particles favor deep penetration of the radiation into the particles. The absorption edges of CdS photocatalysts shift slightly from 511 nm to 505 nm as the temperature used in the solvothermal synthesis increases, indicating the existence of a small quantized transition [18] because it is known that bulk CdS displays an absorption edge at about 515 nm [31,32]. This slight blue shift can be attributed to the elimination of trap energy levels such as structural defects and shallow impurity energy levels associated to the increase in crystallinity observed for the samples prepared at higher solvothermal temperature [33].

### 2.2. Photocatalytic Activity

**Figure 8:**
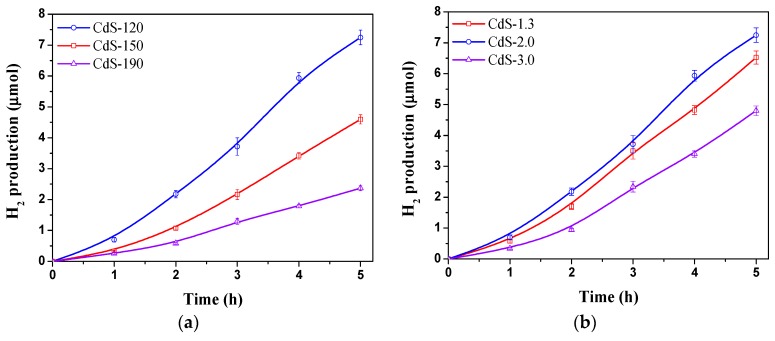
Hydrogen production on CdS photocatalysts: (**a**) influence of solvothermal temperature (CdS-120, 150 and 190); (**b**) influence of H_2_O/thiourea ratio (CdS-1.3, 2.0 and 3.0) (150 W Xe lamp, 0.05 g CdS photocatalyst, 150 mL aqueous solution 0.05 M Na_2_S/0.02 M Na_2_SO_3_).

Figure 8 displays hydrogen production over CdS samples prepared with different solvothermal temperatures and water/thiourea ratios. Significant differences in activity are observed for the CdS samples depending on the solvothermal temperature used. As Figure 8 shows, hydrogen production was found to decrease following the sequence: CdS-120 > CdS-150 > CdS-190. Hydrogen production over CdS samples is also influenced by the water/thiourea ratio used in the solvothermal synthesis. As Figure 8 indicates, hydrogen production was found to decrease following the sequence: CdS-2.0 > CdS-1.3 > CdS-3.0. The decrease in activity observed for CdS samples synthesized with water/thiourea ratio higher than 2.0 contrasts with the increase in activity observed over CdS when the water/thiourea ratio rises from 1.3 to 2.0.

## 3. Discussion

It is well known that the nanostructure morphology and surface characteristics of CdS are determined by the solvothermal conditions used in its preparation [34]. The mechanism of formation of CdS nanostructures in the presence of ethylenediamine (EDA) is determined by: (1) the coordination of ethylenediamine with Cd^2+^ ions forming a stable [Cd(EDA)_2_]^2+^ complex (Equation (1)); (2) the thiourea hydrolysis to generate the S^2−^ ions (Equation (2)); (3) the reaction of [Cd(EDA)_2_]^2+^ complex with S^2−^ ions to form two dimensional CdS(EDA)_m_ structures (Equation (3)) and finally (4) the EDA molecules are eliminated from the unstable CdS(EDA)_m_ structure resulting in the crystallization of CdS (Equation (4)) [26,35]:

(1) $${\text{Cd}}^{2+}+2\left(\text{EDA}\right)\to {\left[\text{Cd}{\left(\text{EDA}\right)}_{2}\right]}^{2+}$$

(2) $${\left({\text{NH}}_{2}\right)}_{2}\text{CS}+2H_{2}O\to 2{\text{NH}}_{3}+H_{2}S+{\text{CO}}_{2}$$

(3) $$\text{Cd}{\left(\text{EDA}\right)}_{m}^{2+}+S^{2-}\to \text{CdS}{\left(\text{EDA}\right)}_{m}$$

(4) $$\text{CdS}{\left(\text{EDA}\right)}_{m}\to \Delta T\to {\left(\text{CdS}\right)}_{m-n}+n\left(\text{EDA}\right)$$

The nanomorphology of CdS during the solvothermal synthesis is controlled by the thermodynamics and kinetics during the nucleation and growth of nanocrystals which in turn are determined by the temperature and the water/thiourea ratio used in the synthesis. Shape tuning of CdS by the controlled addition of sulfide was reported by Shanmugapriya *et al.* [36] who showed the transition from nanospheres to nanorods on increasing the rate of addition of sulfide anions. In line with this, the increase in the temperature during the solvothermal synthesis leads to an increase in the decomposition kinetics of thiourea that increases the release rate of S^2−^ producing both higher rate of crystalline nucleation of CdS and a higher rate of crystal growth. The higher rate of crystalline nucleation induces a higher diameter of the CdS nanostructures since the radial growth mainly occurs in the initial stage of nucleation and it determines the diameter of the formed nanostructures. On the other hand, the rapid crystal growth associated with the increase in temperature implies higher growth along the [001] plane. The increase in crystallinity, diameter and aspect ratio of the CdS samples with the increase on the solvothermal temperature from 120 to 190 °C is consistent with this mechanism. On the contrary, the increase in the release rate of S^2−^ associated to the increase in the decomposition kinetics of thiourea derived from the use of higher water/thiourea ratio implies small increase in the crystallinity and size of the nanorods of CdS formed.

**Figure 9:**
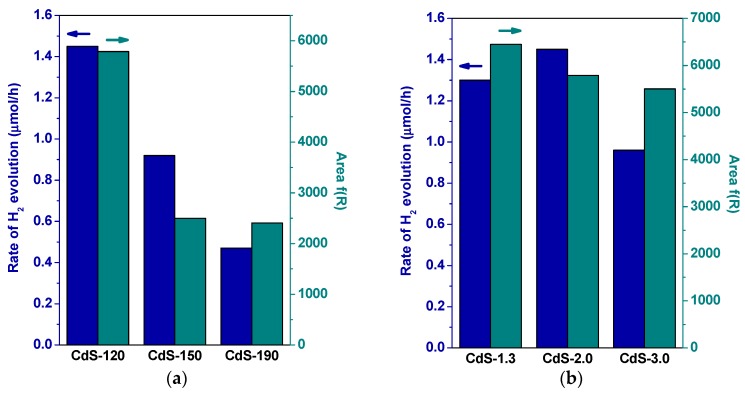
Comparison between hydrogen production rate and integration of UV-Vis absorbance below 500 nm of CdS photocatalysts: (**a**) influence of solvothermal temperature (CdS-120, 150 and 190); (**b**) influence of H_2_O/thiourea ratio (CdS-1.3, 2.0 and 3.0).

The changes in the photocatalytic activity of CdS samples (Figure 8) should be a consequence of their changes in the crystallinity, morphology and size because these parameters affect the generation, separation and migration of the photogenerated charge carriers (e^−^/h^+^) responsible for photoactivity [4,37,38]. With respect to photophysical characteristics of CdS samples, their UV-Vis absorption spectra (Figure 7) showed that the position of absorption edges and the band gap values of all samples (Table 1) were quite similar as a consequence of their similar composition and structure, which are the main factors that determine the band gap of the CdS photocatalyst. Conversely, the absorbance intensity at a wavelength of less than 500 nm showed differences between the CdS samples, in particular in samples synthesized at different solvothermal temperature. Comparison of hydrogen evolution from CdS samples with the integration of absorbance below 500 nm from UV-Vis data (Figure 9) indicated that absorbance capacity of samples is a key parameter in photoactivity because higher absorbance on CdS-120 sample implies higher photoactivity. Nevertheless there is not a parallel behavior between the photoactivity and the absorbance capacity on the rest of the samples, indicating the influence of characteristics other than photophysical properties in these samples.

**Figure 10:**
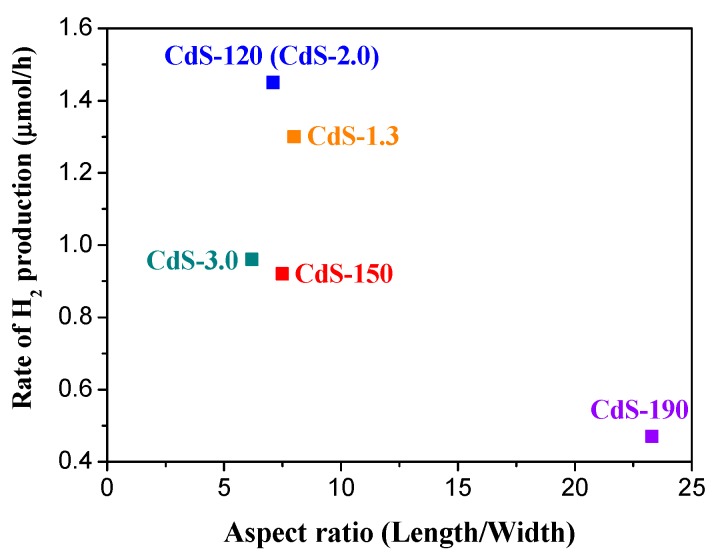
Comparison between hydrogen production rate and the aspect ratio (length/width derived from TEM) of the CdS samples.

Apart from the absorption of photons and the photogeneration of charge carriers (e^−^/h^+^), the separation and migration of these carriers must also be examined in order to justify the differences in the photoactivity of the CdS samples. 1-D nanostructures with dimensions close to the exciton Bohr radius of CdS (2.5 nm) have positive advantages in photochemical reactions because photoexcited electrons and holes are delocalized along the length of the nanostructure but restrained in the radial direction. Thus, recombination of photoexcited electron-holes is slowed down, thereby improving the photophysical processes. Figure 10 shows the comparison between the 1-D aspect ratio of CdS samples obtained by TEM with the photocatalytic hydrogen production. A clear decrease in activity for the CdS sample with higher average aspect ratio (CdS-190 with particle size far to the exciton Bohr radius of CdS) was observed, but the rest of the samples did not show a direct correlation between the aspect ratio and photoactivity indicating the influence of characteristics other than the 1-D aspect ratio on the photoactivity of the CdS nanostructures.

**Figure 11:**
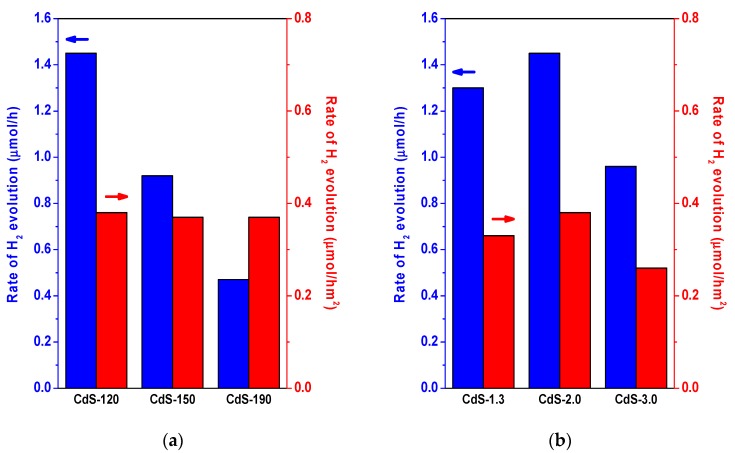
Surface-normalized hydrogen evolution rate (µmol/h m^2^) over the CdS photocatalysts: (**a**) influence of solvothermal temperature (CdS-120, 150 and 190); (**b**) influence of H_2_O/thiourea ratio (CdS-1.3, 2.0 and 3.0).

The surface area of CdS photocatalysts is also a parameter to be taken into account when analyzing the photoactivity behavior of CdS samples. Figure 11 represents the rate of hydrogen production normalized per surface area in order to extract possible influence of surface/bulk structural changes upon the photoactivity of CdS samples prepared at different temperature and H_2_O/thiourea ratio.

As shown in Figure 11a, the CdS samples synthesized at different temperatures presented similar surface-normalized hydrogen production rates. This fact indicated that the increase in crystallinity and size observed on samples synthesized at higher temperature implying lower density of defects does not result in higher photoactivity because these features are compensated by the proportional decrease in surface area. Therefore, the synthesis of nanorods of small size and high surface area exemplified by samples prepared at lower temperature leads to higher photoactivity per mass of photocatalyst because the improvement associated to surface area prevails over the lower recombination of e^−^/h^+^ associated to the higher crystalline degree of the CdS nanostructures obtained at higher temperatures during the solvothermal synthesis. The rate of hydrogen production normalized per surface area on CdS photocatalysts synthesized at H_2_O/thiourea ratios of 1.3 and 2.0 (Figure 11b) showed a similar surface-normalized hydrogen production rate value while this production rate decreased for the sample prepared with H_2_O/thiourea ratio of 3.0. From characterization results of the samples prepared with different H_2_O/thiourea ratios it was observed that the increase in the H_2_O/thiourea implies a monotonically increase in crystallinity and size and a decrease in the nanorod length. By comparing these characteristics with the hydrogen production rate normalized per surface area (Figure 11b) no direct correlation was found, indicating that the decrease in surface-normalized production rate on CdS sample prepared with higher water concentration is not associated to the change of these parameters. Changes at the surface level of CdS in the sample prepared with higher water concentration may play a role in the lower normalized photoactivity observed in this sample. In this sense the lower surface coordination of sulfur in CdS sample prepared with the higher concentration of water, in agreement with TXRF analysis in Table 1, could be the origin of the low photoactivity of this sample. The higher concentration of water during solvothermal synthesis could lead to the partial oxidation of CdS surface, forming sulfates or sulfites, that may decrease the adsorption of H_2_O and S^2−^/SO_3_^2−^ on photocatalyst surface preventing its interaction with the generate e^−^/h^+^ carriers [39]. This possibility will be the objective of a further research.

## 4. Materials and Methods

### 4.1. CdS Synthesis

In a typical synthesis, CdS samples were prepared by solvothermal synthesis using a Teflon-lined stainless steel autoclave (125 mL of volume) charged with 0.0104 mol of cadmium nitrate tetrahydrate, (Cd(NO_3_)_2_·4H_2_O), as metal precursor and thiourea (NH_2_CSNH_2_) as sulphur source, using a Cd:thiourea = 1:3 (molar ratio). Subsequently, ethylenediamine (EDA) was added to 80% volume capacity of the autoclave to dissolve the precursors inside. Finally, 0.0208 mol of water was added to perform total hydrolysis of the thiourea. The autoclave was tightly closed, heated in an oven at the selected temperature for 12 h and left to cool down to room temperature. The yellow precipitates were collected by centrifugation, washed with distilled water several times, washed with absolute ethanol to remove the excess of thiourea and solvent, and then dried under vacuum at 70 °C for 2 h. To investigate the effect of the solvothermal temperature on the crystal structure, morphology and photocatalytic performance of CdS photocatalysts, similar experiments were carried out using different solvothermal temperature: 120, 150 and 190 °C. According to the temperature used in the solvothermal synthesis, samples were labeled as CdS-*x* (*x* = 120, 150 and 190). The effect of the generation of sulfide ions by hydrolysis of thiourea on the structure, morphology and photoactivity of CdS photocatalysts was analogously investigated. To achieve this objective three CdS samples were prepared with different water/thiourea ratio: 1.3, 2 and 3. According to the water/thiourea ratio used in the solvothermal synthesis, samples were labeled as CdS-*x* (*x* = 1.3, 2.0 and 3.0). CdS-120 and CdS-2.0 are the same sample.

### 4.2. CdS Characterization

The chemical composition of CdS samples was determined by total reflection X-ray fluorescence (TXRF) analysis performed with a benchtop S2 PicoFox TXRF spectrometer from Bruker Nano GmbH (Berlin, Germany), equipped with a molybdenum X-ray source working at 50 kV and 600 μA, a multilayer monochromator with 80% reflectivity at 17.5 keV (Mo Kα), an XFlash SDD detector with an effective area of 30 mm^2^, and an energy resolution better than 150 eV for Mn Kα. The fine beam impinges on a polished sample carrier at a very small angle (<0.1°) and is totally reflected. Because the intensity of incident X-ray beams is reflected almost entirely, the remaining intensity penetrates only a few nanometers (approximately 10–15 nm) in the sample.

The specific surface areas of the samples were calculated by applying the BET method to the N_2_ adsorption/desorption isotherms recorded at the liquid nitrogen temperature (−196 °C) on a TRISTAR 3000 instrument (Micromeritics, Norcross, GA, USA) using samples previously degassed under vacuum (*ca.* 10^−4^ mbar) at 120 °C for 2 h to remove all moisture and adsorbed gases on the surface of the sample. The specific surface area values of the samples were calculated by applying the BET equation to the nitrogen adsorption isotherm within the relative pressures 0.05 < *P/P*_0_ < 0.30. Desorption data were used to determinate the pore size distribution by the Barret-Joyner-Halenda (BJH) method, assuming a cylindrical pore model.

The surface morphology and size of the CdS aggregates were observed by Field Emission Scanning Electron Microscopy (FE-SEM) using a XL30 S-FEG Microscope (Philips, Eindhoven, The Netherlands). The morphological characteristics of CdS nanostructures were obtained by transmission electron microscopy (TEM) and high-resolution transmission electron microscopy (HRTEM) with a 2100F TEM/STEM system (JEOL, Tokyo, Japan) operating at 200 kV accelerating voltage with a Field Emission Gun (FEG), obtaining a point resolution of 0.19 nm.

X-ray diffraction patterns of CdS samples were recorded using an X’Pert Pro polycrystal diffractometer (PANalytical, Egham, England) with an X’Celerator RTMS detector and nickel-filtered Cu Kα_1_ radiation (λ = 0.15406 nm, 45 kV, 40 mA) under constant instrument parameters. For each sample, Bragg angles between 4° and 90° (2θ) were scanned with a step size of 0.0335° that was used during a continuous scan in the abovementioned range. Volume-averaged crystallite sizes were determined by applying the Debye-Scherrer equation.

The UV-vis spectra of CdS samples were measured on a Cary 5000 UV-Vis-NIR spectrometer (Varian, Palo Alto, CA, USA) with double beam and double shutter synchronized electronically. The sources are deuterium (UV) and halogen quartz. The detectors were a multiplier and PbS detector refrigerated for the NIR area. Band gap size was obtained from Tauc plots by plotting a tangent line over the slope of the UV-vis spectra and prolonging it to *ƒ(R) = 0*. The wavelength value obtained was converted to [40] given *E_ph_ = hc/λ*, where *E_ph_* is the photon energy, *h* is the Planck constant, *c* the speed of light and *λ* the photon wavelength.

### 4.3. Photocatalytic Activity

The hydrogen evolution from CdS samples was evaluated in a closed Pyrex glass reactor (250 mL total volume, 8 cm diameter) working at room temperature and under Ar atmosphere (0.1 bar). The photocatalyst powders (0.05 g) were dispersed by magnetic stirring in an aqueous solution (150 mL) containing 0.05 M Na_2_S and 0.02 M Na_2_SO_3_ as sacrificial electron-donor agents [41]. Solution pH is 12.5. Using this mixed solution, the photocatalytic reaction should proceed according with the mechanism porposed by Reber and Meier [42] for the photocatalytic hydrogen production from solutions containing mixtures of S^2−^ and SO_3_^2−^:

Photocatalysts + hν → e^−^ (CB) + h^+^ (VB) (5)

2 H_2_O + 2 e^−^ (CB) → H_2_ + 2 OH^−^ (6)

SO_3_^2−^ + H_2_O + 2 h^+^ (VB) → SO_4_^2−^ + 2 H^+^ (7)

2 S^2−^ + 2 h^+^ (VB) → S_2_^2−^ (8)

S_2_^2−^ + SO_3_^2−^ → S_2_O_3_^2−^ + S^2−^ (9)

SO_3_^2−^ + S^2−^ + 2 h^+^ (VB) → S_2_O_3_^2−^ (10)

the overall reaction being:

3 Na_2_(SO_3_) + 2 Na_2_S + 5 H_2_O +6 e^−^ + 6 h^+^ → 3 H_2_ + 4 NaOH + Na_2_SO_4_ + 2 Na_2_(S_2_O_3_) (11)

The reactor was irradiated with a Xe arc lamp (150 W, ozone free, LOT Oriel GmbH & CO KG, Darmstadt, Germany) which includes both UV and visible light. Before measurement, the solution was purged several times with Ar to ensure complete air removal. Samples of the evolved gases were extracted periodically (every 1 h for a total reaction time of 5 h) and analyzed by GC with TCD (Model Star 3400 CX chromatograph, Varian) equipped with a 5Å molecular sieve packed column using Ar as carrier gas.

## 5. Conclusions

CdS nanorods and nanowires with different sizes obtained by solvothermal synthesis were investigated as photocatalysts for hydrogen evolution from solutions containing mixtures of S^2−^ and SO_3_^2-^. The temperature and water/thiourea ratio used in the solvothermal synthesis determine the surface area, shape, length and degree of crystallinity of the CdS nanostructures obtained. The increase in the solvothermal temperature from 120 °C to 190 °C favours the formation of nanorods with higher crystallinity, greater length and width and smaller surface area that finally transform into nanowires of high length and crystallinity at 190 °C. CdS samples synthesized at different temperatures presented similar surface-normalized hydrogen production rates. The change in the water/thiourea ratio affects the crystallinity and length of CdS nanostructures to a lesser extent than temperature. Nevertheless an increase in the water/thiourea ratio used during the solvothermal synthesis resulted in CdS nanorods with higher crystallinity, lower length/width aspect ratio and lower specific surface area. CdS samples synthesized at H_2_O/thiourea ratios of 1.3 and 2.0 showed similar surface-normalized hydrogen production rate values, while this production rate decreased in the sample prepared with a H_2_O/thiourea ratio of 3.0. It can be hypothesized that lower photocatalytic activity of the CdS prepared with higher H_2_O concentration was a consequence of the lower surface coordination of sulfur in this CdS sample derived from the probable partial oxidation of the CdS surface.

## Figures and Tables

**Table 1:** Surface composition (atomic percentage) from TXRF analyses, specific surface area from N_2_ adsorption-desorption isotherms and band gap from UV-Vis spectra of CdS samples.

CdS Sample	Surface Composition		BET (m^2^/g)	Band Gap (eV)
	Cd (%)	S (%)		
CdS-120	46.1	53.9	75.5	2.49
CdS-150	48.2	51.8	49.7	2.45
CdS-190	46.7	53.3	25.7	2.48
CdS-1.3	45.8	54.2	78.9	2.50
CdS-2.0	46.1	53.9	75.5	2.49
CdS-3.0	46.8	53.2	72.4	2.49

**Table 2:** Structural properties of CdS samples determined from XRD data.

CdS Sample	Crystal Structure	Relative Intensity		D_p_ (nm)
		I_(100)/_I_(002)_	I_(101)/_I_(002)_	
CdS-120	Hexagonal	0.44	0.65	43.5
CdS-150	Hexagonal	0.53	0.78	61.0
CdS-190	Hexagonal	1.04	1.58	98.3
CdS-1.3	Hexagonal	0.40	0.67	38.3
CdS-2.0	Hexagonal	0.44	0.65	43.5
CdS-3.0	Hexagonal	0.42	0.70	45.2

**Table 3:** Morphology and size of CdS samples derived from FE-SEM analysis.

CdS Sample	Morphology	Size (µm)
CdS-120	Sheets with emerging irregular filaments	0.4–6.0
CdS-150	Irregular clusters of filaments	0.7–12.0
CdS-190	Defined filaments	4.0–30.0
CdS-1.3	Sheets with emerging irregular filaments	0.3–4.0
CdS-2.0	Sheets with emerging irregular filaments	0.4–6.0
CdS-3.0	Sheets with defined filaments	0.7–8.0

**Table 4:** Morphological characteristics of CdS samples derived from TEM-HRTEM analysis.

CdS Samples	Average Length (nm)	Average Width (nm)	Aspect Ratio (Length/Width)	Morphology
CdS-120	50	7.0	7.1	nanorods
CdS-150	90	12.0	7.5	nanorods
CdS-190	700	30.0	23.3	nanowires
CdS-1.3	60	7.5	8.0	nanorods
CdS-2.0	50	7.0	7.1	nanorods
CdS-3.0	45	7.2	6.2	nanorods
